# The Marine Hospital Service

**Published:** 1878-02

**Authors:** 


ARTICLE V.

*Council of Equity.*

SEC. I. The president, assisted by the vice-presidents, shall appoint a Council of Equity, consisting of....................active members, alumni of those institutions whose courses of study are the most extensive and thorough, etc., etc.

SEC. II. To this council all applications for membership shall be referred after....................charter members have been enrolled. They shall report the names of those only who have the requirements of the constitution. To this council all questions of ethics shall also be referred, and their decision shall be final, etc., etc.

The treasurer, Dr. E. H. M. Sell (51 W. 35th street, New York), will send to any one by mail the Transactions of the Academy in separate parts, this year on receipt of the price of the same in P. O. order or stamps as follows:

Constitution and By-Laws................................ 15 cents.
President's Address (1877) and Constitution and By-Laws.......................................... 50 "

---

We publish the following communication from an esteemed correspondent with great pleasure, and wish that a still wider exception might be made to the general charge, contained in the editorial referred to, against the system of appointments to civil office:

SAN FRANCISCO, Jan. 8, 1878.

*To the Editor of the Journal and Examiner.*

SIR:—In a very timely editorial in the January number of the *Journal and Examiner*, you make the following statement: "In the marine and pension services, surgeons and assistant-surgeons become such by political preferment." Referring to the former service, I beg to inform you that since the reorganization of the Marine Hospital Service under the able administration of the Surgeon-General, Dr. John M. Woodworth, every applicant for appointment has been obliged to undergo a rigid examination by a Board of Surgeons, with reference to his professional and physical qualifications. The examination, which extends over a period of four days, is both written and oral, embracing all the branches of medicine, together with a clinical examination, both medical and surgical at a hospital. As the examination is strictly competetive, the candidates are appointed according to the highest percentage. No appointment is made to a higher grade than assistant surgeon in the Marine Hospital Service, and all vacancies which occurr in the grade of surgeon, are filled by promotion of assistant surgeons, on the ground of merit and fitness only.

In justice to our service, you will oblige me by affording this communication a space in your valuable journal.

Very respectfully,

EDMUND J. DOERING, M. D.
*Asst. Surgeon U. S. Marine Hospt. Service.*

---

CLINTON, ILL, January, 1878.

*To the Editor of the Journal and Examiner.*

MAY DEAR SIR:—I have thought the enclosed scrap, taken from the *Pittsburgh Gazette*, of July 7th, 1818, might be of sufficient interest to deserve a place in the JOURNAL AND EXAMINER.

Respectfully, C. GOODBRAKE.

INTERESTING DISCOVERY.

In the history of medicine, since the discovery of the circulation cf the blood by Dr. Harvey, we have not heard of any of equal importance, except a discovery lately made in England by Sir Everard Home, which will most probably overturn the whole of the present practice in medicine.

It has been a prevailing idea that a drop of any fluid introduced or injected into any of the veins produces an immediate coagulation of the blood; but Sir Everard Home has ascertained by repeated experiments that medicines directly injected or introduced into the veins produce their effects more rapidly, with more benefit, and with less injury to the system than when they are swallowed and then pass from the stomach into the circulation.

Sir Everard Home has ascertained not only from experiments made upon himself, but upon many others, that a *vinous infusion* of the *colchicum autumnale* or *meadow saffron* injected into the vein of the ankle or the leg, will cure the most violent gout. Sir Everard states that he completely recovered from a most violent attack of the gout in less than twenty hours by injecting into the circulation sixty drops of this medicine.

Sir Everard mentions that infusions of ipecacuanha and jalap injected into the jugular vein, produced their respective effects of vomiting and purging much more rapidly, and with more ease to the patient, than when taken by the mouth.

An infusion of rhubarb, when injected, causes a profuse flow of urine. In short, according to the experiments of Sir Everard Home, all medicines whatever act better, and are less injurious to the constitution, when injected into the veins than when swallowed by the mouth.

Although the reputation of Sir Everard Home in the science of medicine is of the first rank, yet we would wish to have more satisfactory evidence of the effect of his practice before we should recommend a trial of it. We have no doubt that all those sciences immediately connected with the animal and vegetable kingdoms are yet in their infancy, and that great improvements will be made before the *lapse of many years*.

PETER. INTEL.

---

THE attention of medical practitioners in this State, is called to the fact that the Illinois Board of Health has recently issued new blank forms, for the purpose of reporting births (premature and at term), deaths, and cases of contagious disease.

These blanks require more details of fact than the old ones; and should be sent, when filled out, to the office of the county clerk.

